# Foot-and-mouth disease: past, present and future

**DOI:** 10.1186/1297-9716-44-116

**Published:** 2013-12-05

**Authors:** Syed M Jamal, Graham J Belsham

**Affiliations:** 1 Department of Biotechnology, University of Malakand, Chakdara Dir (L), Khyber Pakhtunkhwa, Pakistan; 2 National Veterinary Institute, Technical University of Denmark, Lindholm, 4771 Kalvehave, Denmark

## Abstract

Foot-and-mouth disease (FMD) is a highly contagious disease of cloven-hoofed animals including cattle, pigs, sheep and many wildlife species. It can cause enormous economic losses when incursions occur into countries which are normally disease free. In addition, it has long-term effects within countries where the disease is endemic due to reduced animal productivity and the restrictions on international trade in animal products. The disease is caused by infection with foot-and-mouth disease virus (FMDV), a picornavirus. Seven different serotypes (and numerous variants) of FMDV have been identified. Some serotypes have a restricted geographical distribution, e.g. Asia-1, whereas others, notably serotype O, occur in many different regions. There is no cross-protection between serotypes and sometimes protection conferred by vaccines even of the same serotype can be limited. Thus it is important to characterize the viruses that are circulating if vaccination is being used for disease control. This review describes current methods for the detection and characterization of FMDVs. Sequence information is increasingly being used for identifying the source of outbreaks. In addition such information can be used to understand antigenic change within virus strains. The challenges and opportunities for improving the control of the disease within endemic settings, with a focus on Eurasia, are discussed, including the role of the FAO/EuFMD/OIE Progressive Control Pathway. Better control of the disease in endemic areas reduces the risk of incursions into disease-free regions.

## Table of contents

1. Introduction

2. Structure of FMDV

3. Serotypes of FMDV

4. Diagnosis of FMD

4.1. Neutralization test

4.2. Enzyme linked immunosorbent assay (ELISA)

4.3. Virus isolation

4.4. Reverse transcription-polymerase chain reaction (RT-PCR)

4.5. Reverse transcription loop-mediated isothermal amplification (RT-LAMP)

4.6. Chromatographic strip test

4.7. Differentiation between infected and vaccinated animals (DIVA)

5. Characterization of FMDV below the level of serotype (strains/subtypes)

6. Geographical Distribution of FMD

7. FMD virus pools

8. Progressive control pathway for FMD

9. Conclusions/recommendations

10. Competing interests

11. Authors’ contributions

12. Acknowledgements

13. References

## 1. Introduction

The earliest description of probable foot-and-mouth disease (FMD) in cattle was made by an Italian monk, Hieronymus Fracastorius, in Venice in 1514. The affected animals refused their feed, the oral mucosa showed redness and the animals had vesicles in the oral cavity and on their feet. Most of the affected animals eventually recovered. This description, made 500 years ago, shows a strong resemblance to that of FMD when seen currently. FMD is considered one of the most important diseases of cloven-hoofed animals; it affects cattle, buffaloes, pigs, sheep, goats and about 70 wildlife species, e.g. African buffaloes. The disease has been present in almost every part of the world where livestock are kept. More than 100 countries are still affected by FMD worldwide and distribution of the disease roughly reflects economic development. The more developed countries have eradicated the disease. However, an incursion of the disease into the normally disease-free countries can cause enormous economic losses. The disease is caused by a single stranded positive sense RNA virus, foot-and-mouth disease virus (FMDV), belonging to the genus *Aphthovirus* within the family *Picornaviridae*. This review includes a description of the properties of the virus and the systems for detecting and characterizing FMD outbreaks. This information is then used to describe the current distribution of the disease/virus and how the FAO/EuFMD/OIE Progressive Control Pathway can assist in disease control within endemic countries and hence reduce the risk of incursions into disease free regions. The major focus here is on Eurasia but other regions are also considered when appropriate.

## 2. Structure of FMDV

**Figure 1:**
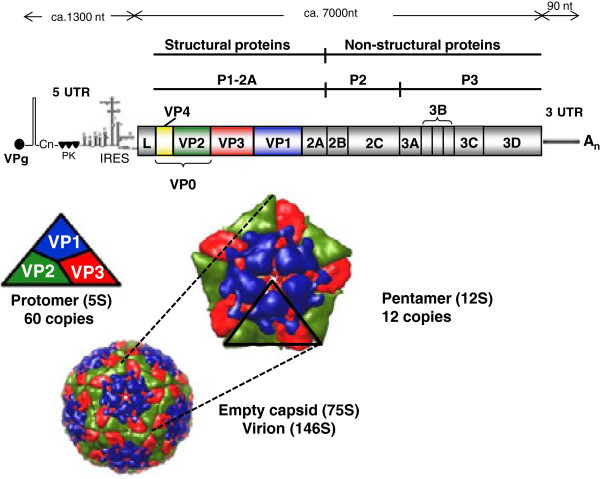
**Genome organization of FMDV and the structure of virus.** The FMDV genome includes a single large ORF, indicated by the shaded rectangle. The regions within the rectangle indicate the individual proteins. The 5’ UTR includes several distinct structural elements including: a poly(C) tract (Cn), 3 or 4 pseudoknots (PK) and the internal ribosome entry site (IRES). The VPg peptide is made in 3 different forms (encoded by the 3B_1-3_) and each acts as the primer for RNA synthesis so each RNA genome, when synthesized, is covalently linked to a VPg. The assembly of virus particles from protomeric and pentameric subunits is indicated. Assembled virus particles contain a single copy of the viral RNA and 60 copies of the 4 different capsid proteins (VP1-VP4). Self-assembly of empty capsid particles, lacking the RNA genome, can also occur. The VP4 protein is internal.

The FMDV particle is roughly spherical in shape and about 25–30 nm in diameter. It consists of the RNA genome surrounded by a protein shell or capsid. The capsid is composed of 60 copies of the capsomers. Each capsomer consists of four structural polypeptides, VP1, VP2, VP3 and VP4. The VP1, VP2 and VP3 are exposed on the surface of the virus while VP4 is located internally. The protein coat surrounds a single stranded, positive sense RNA genome about 8400 nucleotides (nt) in length. The RNA includes three separate parts i.e. the 5′ untranslated region (5′ UTR), a long coding region and the 3′ untranslated region (3′ UTR) (Figure 1). A small protein (24 or 25 residues long), termed VPg, which is encoded by the 3B portion of the viral genome region, is covalently linked to the 5′ end of the genome.

The 5′ UTR is about 1300 nt in length [1] and consists of an S fragment at its 5′ end, a poly C tract, a series of RNA pseudoknot structures, a *cis*-acting replication element (*cre*) (also known as the 3B-uridylylation site (*bus*)), and the internal ribosome entry site (IRES). The S fragment is 360 nt in length and is predicted to fold to form a large hairpin structure. The poly C tract is of variable length (150–250 nt) but is comprised of over 90% C residues. The function of the pseudoknots is unknown. The *cre*/*bus* is a stable stem-loop element of about 55 nucleotides and contains a conserved motif (AAACA) which acts as a template for uridylylation of VPg (3B_1-3_) by the viral RNA polymerase. Thus the *cre*/*bus* is involved in the initiation of RNA replication. The IRES is about 450 nt in length and is responsible for cap-independent initiation of viral protein synthesis [1].

The coding region follows the 5′ UTR. It is the major portion of the viral genome and is about 7000 nt in length. It encodes a large polyprotein which is then cleaved by viral proteases to form four different structural and eleven different non-structural proteins plus a variety of precursors, some of which have distinct functions. After translation, initially four primary products are formed, namely, L^pro^, P1-2A, P2 and P3. The Leader protease (L^pro^) is the N-terminal component of the polyprotein. The L coding region contains two separate AUG initiation codons (usually 84 nucleotides apart) that result in the generation of two different L proteins, termed Lab and Lb. The L^pro^ is responsible for the inhibition of host cell protein synthesis by inducing the cleavage of the host protein, eIF4G, which is a translation initiation factor [1] that is required for the translation of the capped cellular mRNAs. As a result, FMDV RNA can freely use the host cell’s protein synthesis machinery for its own protein synthesis since the FMDV IRES can function with the residual C-terminal fragment of eIF4G [1]. The P1-2A capsid precursor is cleaved by the 3C protease (3C^pro^) to produce 1AB (VP0), 1C (VP3) and 1D (VP1) (plus 2A) and during encapsidation of the genome the VP0 is cleaved to make VP4 and VP2 (note 2A is a very short peptide, <20 residues). The VP4 is entirely internal within the virus particle whereas VP1, VP2 and VP3 are surface exposed and contribute to the antigenic properties of the virus [2,3]. The VP1 contains at least two important immunogenic sites, the G-H loop (at amino acid positions 141–160) and the C-terminus (residues 200–213). The G-H loop includes an arginine-glycine-aspartic acid (RGD) motif, which is required for attachment of the virus to the host cell via an integrin receptor [4,5]. Integrins are a group of α-β heterodimeric glycoproteins which are located on the cell surface; some 15 α and 8 β sub-units combine to form 20 different α-β heterodimers. The αvβ6 heterodimer is a receptor for the extracellular matrix proteins whose expression is restricted to epithelial cells and it also binds to FMDV through interaction with the RGD motif [6]. The virus, however, can also infect cells in an RGD-independent manner using alternative molecules e.g. heparan sulphate proteoglycans receptors [7].

The nucleotide sequences of the VP1 coding region have been used for genetic characterization of FMDV strains because of their significance for virus attachment and entry, protective immunity and serotype specificity. VP1 sequence based phylogenetic analyses have been used widely to deduce evolutionary dynamics, epidemiological relationships among the genetic lineages and in the tracing of the origin and movement of outbreak strains [8-12].

The P2 and P3 regions of the polyprotein are processed to the non-structural proteins (NSPs) [1]. The P2 region generates the proteins 2B and 2C while the P3 region is cleaved to form the proteins 3A, three distinct copies of VPg (3B_1-3_), 3C^pro^ and 3D^pol^. The P2 and P3 encoded proteins are involved in protein processing (3C^pro^) and genome replication (2B, 2C, 3A, 3B_1-3_ (VPg) and 3D^pol^). The 3C^pro^ is responsible for cleavage of P1-2A into VP0, VP1, VP3 as well as the formation of the different non-structural proteins.

The 3′ UTR is much shorter than the 5′ UTR. It is about 90 nucleotides long and folds to form a specific stem-loop structure, followed by a polyA tract of variable length [13]. The 3′ UTR must play an important role in viral genome replication.

## 3. Serotypes of FMDV

FMD virus exists as seven different serologically distinct types. Serotypes O and A were initially discovered by Vallee and Carre [14]. They showed that cattle that had recovered from clinical disease due to an FMD virus which originated in France became re-infected almost immediately when mixed with animals infected with FMD virus that originated in Germany. They named these serotypes after their place of origin; O for the department of Oise in France and A for Allemagne (the French word for Germany). Their work was extended by Waldmann and Trautwein [15] with the discovery of a third serotype which was named serotype C. Later three additional serotypes were identified in samples originating from South Africa and they were named as Southern African Territories 1, 2 and 3 (SAT1, SAT2, SAT3) [16]. The seventh serotype, Asia-1, was initially detected in a sample collected from a water buffalo at Okara, Punjab, Pakistan in 1954 [17]. Examination of an extensive number of samples from across the world have failed to reveal the existence of another serotype although there are many different “sub-types”, some of which are quite distinct from other strains of the same serotype.

## 4. Diagnosis of FMD

Due to the rapidity of spread of FMD and the serious economic consequences that can arise from an outbreak, prompt, sensitive and specific laboratory diagnosis and identification of the serotype of the viruses involved in disease outbreaks is essential. The disease is diagnosed based on clinical signs, including high temperature, excessive salivation, formation of vesicles on the oral mucosa, on the nose plus the inter-digital spaces and coronary bands on the feet. However, the clinical signs can be confused with other diseases (e.g. vesicular stomatitis and swine vesicular disease) and thus laboratory based diagnosis is also necessary. Furthermore, there is no cross protection between the serotypes and the serotype of a virus involved in an outbreak cannot be ascertained on the basis of clinical signs. Thus determination of the serotype involved in field outbreaks has to be established within laboratories to permit proper control/vaccination programs to be followed. Various techniques have been used to diagnose the disease and to ascertain the serotype of the virus. The current methods are described below:

### 4.1. Neutralization test

The virus neutralization test (VNT) is currently considered as the “gold standard” for detection of antibodies to structural proteins of FMDV and is a prescribed test for import/export certification of animals/animal products [18]. However, as various primary cells and cell lines with variable degrees of sensitivities are used in the VNTs, they are more prone to variability than other serological tests. Furthermore, VNT is slower, subject to contamination and requires restrictive biocontainment facilities in contrast to other serological tests which can use inactivated viruses as antigens.

### 4.2. Enzyme linked immunosorbent assay (ELISA)

The complement fixation test (CFT) was the test of choice for diagnosis of FMD and virus typing until the 1970s and is still used in some endemic areas. However, in order to overcome the problems of its low sensitivity and difficulty in interpretation of its results due to pro- and anti-complement activities, enzyme linked immunosorbent assays (ELISAs) for antigen detection and virus typing were desired. Roeder and Le Blanc Smith [19] established suitable assays by using high titre antisera raised in rabbits and guinea pigs against purified 146S FMDV particles for antigen capture and detection, respectively. The assays were found to be 125 times more sensitive than the CFT and are still routinely used for the diagnosis of FMD and for virus typing. The ELISA, however, gives positive results with only about 70-80% of epithelial suspensions that contain virus due to a lack of sensitivity. Thus the virus may have to be propagated in tissue culture and subsequently tested in ELISA to detect the virus and ascertain the serotype.

Monoclonal antibody (MAb)-based ELISAs have also been developed for diagnosis of FMD and virus typing [20,21]. Recently, a sandwich ELISA using recombinant integrin αvβ6 (a receptor for FMDV) for virus capture and serotype-specific monoclonal antibodies as detecting reagents was compared with the conventional polyclonal antibody-based sandwich ELISAs for the identification and serotyping of all the seven types of FMDV. The integrin/MAb ELISA recognized FMDVs of wide antigenic and molecular diversity from all seven serotypes. Although some FMDVs could not be detected, the assay showed greater specificity than the conventional polyclonal ELISA while retaining test sensitivity [22].

### 4.3. Virus isolation

As indicated above, the presence of relatively high levels of FMDV antigen in vesicular material can be detected by ELISA. However, when the virus concentration is too low to be detected by ELISA, then it has to be propagated in susceptible cell cultures. Primary cell cultures (such as bovine thyroid cells and porcine or ovine kidney cells) or cell lines (such as BHK or IBRS2) are considered to be generally suitable for isolation of FMDV [18]. However, the production of consistent quality, ready-for-use primary cells is laborious, time-consuming and expensive. Furthermore, virus isolation requires the presence of infectious virus, which depends on sample quality. Up to 4 days may be required to demonstrate the presence of virus, especially when the levels of virus are low (thus it also takes 4 days to be confident, using this methodology, that no virus is present). Moreover, some FMDVs fail to grow in a specific cell type. Thus the absence of apparent growth does not guarantee absence of the virus and therefore samples collected from a suspected case of FMD should be subjected to further investigations, e.g. using another testing system. Additional disadvantages include the problems associated with obtaining and maintaining a regular supply of cells; possible contamination of cell cultures and the necessity to confirm any apparent virus growth by ELISA. These issues may delay the initiation of control measures to contain outbreaks.

### 4.4. Reverse transcription-polymerase chain reaction (RT-PCR)

The reverse transcription-polymerase chain reaction (RT-PCR) has been shown to be a useful tool for the diagnosis of FMD as it offers the advantages of fast, sensitive and reliable diagnosis. A variety of RT-PCR methods have been reported in recent years for the early detection of FMDV RNA in epithelium, cell culture isolates and other tissues using universal primers for all seven serotypes [23]. Typing of FMDV by RT-PCR was first demonstrated by Rodriguez et al. [24] for the differentiation of the serotypes O, A and C. Serotype specific primers have since been designed for the detection of all seven FMDV serotypes by RT-PCR [25,26]. Primers designed for these assays target various regions of the virus genome, including the 5′ UTR, the open reading frame and the 3′ UTR. However, evaluation of available sets of primers, designed for universal and serotype-specific diagnosis of FMDV, on a variety of field samples, representing all the seven serotypes of FMDV, has shown that no single primer set is capable of diagnosing the disease or typing of the virus. In order to improve the diagnostic sensitivity of RT-PCR, multiplex assays, incorporating more than one set of primers have been developed [27,28]. However, differentiation/serotyping could only be made for certain groups of serotypes or individual isolates. Thus the conventional RT-PCR is not sufficiently sensitive and specific to replace methods using virus propagation in cell culture and ELISA.

Recently, real time/quantitative RT-PCR (rRT-PCR) methods have been developed which do not require post-PCR processing (e.g. gel analysis) and the signals can be monitored directly as the target cDNA is being amplified. Other advantages of the rRT-PCR include high throughput capability and the ability to quantify the genetic material in the starting sample. A TaqMan assay has been shown to be very robust and as effective for primary detection of FMDV as virus isolation in conjunction with antigen ELISA [29]. Currently, two different rRT-PCR TaqMan assays are in common use, one targeting the internal ribosomal entry site (IRES) within the 5′ UTR [30] and the second targeting the 3D (RNA polymerase) coding sequence [31].

The speed and accuracy of detection of the rRT-PCR assay was further improved by coupling the assays with robotic methods for extraction of nucleic acid from the samples and for set up of the assays. This has made the assay highly suitable for the diagnosis of the primary index case and for use in an ongoing outbreak. The rRT-PCR assays are currently used as a routine test for FMD diagnosis and quantification of the virus in many developed countries. However, these assays are not designed to discriminate between serotypes of FMDV. Although these assays exploit highly conserved regions across all the seven serotypes of FMDV, serotype biases still exist within both of these assays. The 5′ UTR assay has been shown to be more sensitive in detecting serotype A viruses, whereas, the 3D assay has greater sensitivity for detecting the SAT viruses [32]. Additionally each of the assays failed to detect a small number of FMDV isolates due to the presence of nucleotide mismatches within the region targeted by the probes. Thus, no single stand-alone assay is capable of detecting FMDV with 100% sensitivity. Recently, Tam and colleagues [33] reported fluorescence-based multiplex rRT-PCR assays for the detection of FMDV and virus typing. The assay was found to have greater sensitivity for detection but some cross-reactivity between some serotypes was also noted. Further work is in progress to develop rRT-PCR assays for serotyping of the virus.

### 4.5. Reverse transcription loop-mediated isothermal amplification (RT-LAMP)

The development of portable equipment for rRT-PCR has enabled molecular diagnosis of FMD possible in the field. This approach, however, requires expensive and fragile instruments and relies on precision thermocycling. Thus other approaches, like loop-mediated amplification (LAMP), were developed, which enable the tests to be conducted in the field using inexpensive tools. LAMP amplifies specific nucleotide sequences at a constant temperature and thus does not require a thermocycler. The assay is based on the principle of DNA amplification by an autocycling strand displacement reaction. The assay is performed using a set of two specially designed inner primers and two outer primers and a DNA polymerase with high strand displacement activity [34]. The primers recognize 6 independent target sequences in the initial stage and 4 independent sequences during the later stages of the LAMP reaction. The reaction is carried out in less than an hour using a standard water bath or heating block and the results can be visualized with the naked eye. The advantages of its simple operation, rapid reaction and potential for visual interpretation without instrumentation make the technique attractive for field use in endemic countries. The RT-LAMP assay that has been developed for FMDV detection can be used in a high throughput system [35]. This assay has, however, not yet been extensively evaluated for its ability to replace or supplement the techniques currently in use.

### 4.6. Chromatographic strip test

Virus isolation combined with ELISA and RT-PCR assays are reliable and accurate for the diagnosis of FMD but the shipment of samples from the field to the laboratory and the poor quality/amount of submitted samples can result in hindrance of early diagnosis of the disease. A rapid and specific test for disease diagnosis at the site of a suspected outbreak may allow timely implementation of control measures. A MAb-based chromatographic strip test for FMD diagnosis was developed by Reid et al. [36]. The test was found to be at least as sensitive as the conventional antigen ELISA for the detection of FMDV in epithelial suspensions tested and had an equivalent 100% sensitivity on the cell culture supernatants of FMDV serotypes O, A, C and Asia-1. Further research is underway to develop chromatographic strip tests capable of ascertaining the serotype of the virus.

### 4.7. Differentiation between infected and vaccinated animals (DIVA)

Detection of animals that have been infected with FMDV is of considerable importance for the control of FMD especially in a previously FMD free country or in a country with sporadic outbreaks. Both previously infected and vaccinated animals can have neutralizing antibodies in their sera, but it is important for trade purposes to be able to distinguish previously infected animals from those that have just been vaccinated against the disease. This is because a high proportion (up to 50%) of animals infected with FMDV can become “carriers”, these are defined as animals which continue to have infectious virus present within the oropharynx more than 28 days post-infection. The animals are clinically normal and can maintain this state for a long period (ca. 2–3 years in cattle). It is possible that such animals can act as a source of infection for other animals, indeed evidence for this process exists for African buffalo, however it has not been demonstrated in experimental studies with cattle alone (see [37] and [38] for more detailed descriptions). Viral replication during infection results in the production of both structural (SP) and nonstructural (NSP) proteins. Like the SPs, some NSPs are immunogenic [39]. Vaccines consisting of purified preparations of inactivated 146S virions induce antibodies almost exclusively against the SP of the virus (at least after a small number of vaccinations). Thus it can be possible to discriminate between infected and vaccinated animals based on the detection of antibodies to NSPs.

Differentiation of infection from vaccination based on the antibodies to NSP has been reported using either panels of proteins or the individual proteins 2C or 3ABC. Early assays for the detection of anti-NSP antibodies relied on radioimmunoprecipitation [40] or enzyme linked immunoelectrotransfer blot assays [41]. However, these assays are not suited for rapid examination of large numbers of sera and thus alternative techniques like ELISA were developed. Several ELISAs based on the detection of antibodies to various NSPs of FMDV have been established [42,43]. However, these ELISAs used species-specific conjugates, making simultaneous examination of sera from different species difficult. Thus there was a need for an assay which enabled simultaneous testing of sera from different species. Sørensen et al. [44] developed a blocking ELISA which was species independent; using baculovirus expressed FMDV NSPs as antigen and polyclonal antibodies produced in guinea pigs as capture and detecting antibodies. The polyclonal antibodies were later replaced with monoclonal antibodies [45] for high throughput. As antibodies to NSP persist for long periods [46], positive animals are not necessarily still infected although they can be carriers (see above). However, tests for detection of NSP antibodies cannot be used for detection of carrier animals as some persistently infected animals do not show sero-conversion against NSPs [47], the carrier animal status may occur in previously vaccinated animals in which only limited virus replication occurs. Moreover, no serological tests are currently available that can differentiate between FMDV carriers and other animals that show a serological response to FMDV. Furthermore, it can be difficult to differentiate between vaccinated and previously-infected animals if the vaccine used has been prepared from cell culture supernatant (i.e. not purified) and/or contains varying degrees of contaminating viral NSPs especially if multiple vaccinations have occurred.

## 5. Characterization of FMDV below the level of serotype (strains/subtypes)

Within each serotype of FMDV, there is a spectrum of variants with their own antigenic, biological and epidemiological characteristics. In some cases there is poor cross-protection between variants within a serotype and thus characterization of sub-serotypes/strains becomes necessary to ensure selection of appropriate vaccines to control an outbreak. Initially strains were characterized based on their performance in cross-protection tests in animals. Subtype variants were distinguished by the fact that immunization against one subtype did not confer the same level of immunity to another variant of the same serotype as to the homologous strain. Animals vaccinated with one strain withstood homologous challenge but were only partially protected against challenge with a heterologous strain [48,49]. Quantitative cross-protection tests based on determination of heterologous and homologous 50% protective dose (PD_50_) values have been derived [50]. However, such tests are very costly for routine use, time-consuming and are subject to considerable variation due to differences in individual animal susceptibility. Thus other methods have been developed.

Antigenic characterization was used to compare field viruses with vaccine strains by determination of the serological relationship (r_1_ value) using hyperimmune sera in ELISA [51] or in VNTs using cell culture [52]. Using VNTs, r_1_ values of ≥ 0.3 have been shown to reflect a close antigenic relationship between the field isolates and vaccine strains, indicative of good protection by the vaccine, whereas values < 0.3 reflect a more distant antigenic relationship, indicating that the vaccine is unlikely to protect against the field isolates. Antigenic characterization using serological tests like VNT and ELISA, using defined sera/MAbs, are useful in showing antigenic diversity but they are unable to characterize strains individually and cannot be used to trace the origin of an outbreak.

Nucleotide sequence analysis has now become the definitive technique for characterization of FMDV strains. This technique was first used for the study of the epidemiology of FMD by Beck and Strohmaier [53], who investigated the origin of FMDV outbreaks in Europe over a 20 year period. The first phylogenetic analysis of FMDV using nucleotide coding sequences for VP1 was reported by Dopazo et al. [54]. Since then a number of studies have been published on nucleotide sequence analysis for all the seven serotypes of FMDV (e.g., [8,9,12,55,56]), some of these have used complete genome sequences for tracing of outbreaks [8,12].

The serotypes of FMDV have on average 86% nucleotide sequence identity to each other across the whole genome [57] but the VP1 coding region is substantially more variable and shows only about 50-70% identity [58]. Serotypes O, A, C and Asia-1 have been further classified into genotypes based on up to 15% difference in VP1 coding sequences [9,58]. Thus, FMD serotype O viruses from around the world have been classified into eight genetically and geographically distinct genotypes, called topotypes, on the basis of the VP1 coding sequence [11,58]. The eight topotypes are Middle East-South Asia (ME-SA), South–East Asia (SEA), Cathay, Indonesia-1, Indonesia-2, East Africa, West Africa and Europe-South America (Euro-SA). Among these, the first five are circulating in Asia, of which the dominant one is ME-SA [58]. Four different lineages within the ME-SA topotype have been defined on the basis of phylogenetic relationships and a nucleotide sequence difference (within the VP1 coding sequence) of > 7.5% [58,59]. Within the ME-SA topotype, a pandemic strain of FMDV, designated as the PanAsia lineage, spread vigorously and is discussed in detail below.

FMDV serotype A is genetically and antigenically the most diverse of the Eurasian serotypes [58]. Up to 32 sub-types had been defined by the late 1970s [60,61] though this system of sub-typing was later discontinued. More recently, serotype A FMDVs, from the whole world, have been classified into 26 genotypes based on > 15% difference in VP1 coding sequence [62]. The serotype A viruses form three geographically distinct genotypes (topotypes) i.e. Asia, Europe-South America (Euro-SA) and Africa. The level of nucleotide differences, within the VP1 coding region, between serotype A viruses belonging to different continental topotypes is up to ~24% [62]. Topotype Asia is the most prevalent in the Middle East and South Asian region and exists in various lineages e.g. A_15_, A_22_, A-IRN87, A-IRN96, A-IRN99, A-Iran05, etc. The A-Iran05 lineage is currently dominant in the West Eurasian region and has evolved into different sublineages [9,63]. Viruses belonging to the A-Iran05^BAR-08^ sublineage are antigenically distinct from the vaccine strain, A22/Iraq [9].

FMDV serotype Asia-1 is considered to be genetically and antigenically the least diverse serotype [58]. Indeed, Ansell et al. [64] reported that 44 serotype Asia-1 FMDVs isolated between 1954 and 1990 throughout Asia were less variable compared to other FMDV serotypes in their VP1 coding sequences. It is noteworthy, however, that the RGDLXXL receptor binding motif of serotype Asia-1 viruses was more variable when compared to serotypes O and A of FMDV [9,56,65]. Previous reports have classified FMDV serotype Asia-1 in different ways. For example, Ansell et al. [64] grouped serotype Asia-1 viruses isolated throughout Asia between 1952 to 1992 into 18 groups, while Mohapatra et al. [66] classified serotype Asia-1 viruses from India, sampled over the last two decades, into seven lineages and Valarcher et al. [67] divided these FMDVs from 2003–2007 into six Groups. An additional Group of Asia-1 viruses, designated as Group-VII [56] and later named as Sindh-08 by WRL-FMD, has also been recently reported, which is currently circulating in the West Eurasian region. Viruses belonging to the Group-VII are not efficiently neutralized by antisera raised against the Asia-1/Shamir and Asia-1/Ind/8/79 vaccine strains [56]. Outbreaks due to this novel Group have also been reported in animals given vaccine based on the Asia-1/Shamir strain. A homologous vaccine prepared from an isolate (Asia-1/TUR/ 11) responsible for a field outbreak in Turkey in 2011, has been found to be effective to contain the spread of the Group-VII (Sindh-08) viruses [68].

Unfortunately, there are no uniform criteria or nomenclature for these classifications. As indicated, serotypes O and A FMDVs have been classified into lineages but serotype Asia-1 FMDVs have been classified into Groups (see [56,67]).

## 6. Geographical distribution of FMD

The serotypes of FMDV are not distributed uniformly around the world. The serotype O, A and C viruses have had the widest distribution and have been responsible for outbreaks in Europe, America, Asia and Africa. However, the last reported outbreak due to serotype C FMDV was in Ethiopia during 2005 [69] and so serotype C viruses may no longer exist outside of laboratories. The SAT1-3 viruses are normally restricted to sub-Saharan Africa. However, there have been some limited outbreaks due to SAT1 viruses in the Middle East between 1962–1965 and 1969–1970 and then in Greece in 1962 [58]. Similarly, there have been reports of minor incursions of the serotype SAT2 in Yemen in 1990 and in Kuwait and Saudi Arabia in 2000 [70]. More recently, FMD outbreaks due to serotype SAT2 spread from sub-Saharan Africa through northern African countries (Egypt and Libya) and into Palestine [71]. This serotype was also detected in Bahrain. The serotype Asia-1 FMDVs are generally confined to Asia, except for two incursions into Greece, one in 1984 and a second in 2000.

Although considerable information is available on the virus, the disease and vaccines, FMD still affects extensive areas of the world. FMD free countries have introduced a number of measures to retain their status because of the heavy economic losses resulting from this disease. The USA has experienced FMD nine times since 1870. Each time the disease was eradicated with strict slaughter and quarantine control procedures. The last FMD outbreak in the USA occurred near Montebello, California, in 1929. Infected hogs contracted the disease after being fed swill with meat scraps from a tourist steamship coming from Argentina. Since then, the USA has had restrictions on importation of susceptible animals and livestock products from countries where FMD is present. Canada has been free of FMD since 1952 and Mexico since 1953.

Historically, FMD in Australia was documented in the early 1800s and early 1870s [72]. In 1871–1872, at least five episodes of FMD were documented in cattle that originated from the UK, either bound for Australia, in quarantine or in a bull which had landed two months earlier [72]. The last outbreak of FMD in Australia was reported in 1872.

The Pandemic serotype O virus (designated as the PanAsia strain) belongs to the ME-SA topotype which has spread rapidly and vigorously [73]. This lineage replaced the other lineages of FMDV previously circulating in the Middle East [74]. This lineage has been responsible for disease outbreaks everywhere in the world where FMD is endemic or sporadic except for South America and been responsible for incursions into previously disease-free countries. The PanAsia lineage was first detected in India in 1982 [59] but was confined to India until 1990. Its predominance in field outbreaks in India was, however, noticed from 1996 onwards [59]. It spread northwards to Nepal in 1990 and again in 1997–1999 and to Bhutan in 1998 and also towards the west, into Bahrain, Kuwait, Saudi Arabia, Syria, Yemen, Iran and Lebanon in 1998 and to UAE, Israel and Turkey in 1999 [73]. The lineage spread further to China in 1999 and into South East Asian countries causing outbreaks in Thailand in 1999, Malaysia and Laos (PDR) in 2000 and Vietnam in 2002. The virus also caused disease outbreaks in South Korea [75] and Japan in 2000 [76]. These two countries were previously free of FMD since 1934 and 1908, respectively. South Korea faced outbreaks again in 2002 and then in 2010. The 2002 outbreaks were caused by serotype O virus, belonging to the PanAsia lineage, whereas, both serotype O (topotype SEA, lineage MYA-98) and A (topotype ASIA, genotype SEA, lineage MYA-97) viruses were responsible for the 2010–2011 outbreaks [77]. South Korea appears to have had 3 independent introductions of the virus in 2010. Firstly there was an incursion of FMDV serotype A (A/SEA/MYA/97) in January, 2010. The disease was controlled using a stamping out policy [78]. There had been no reported outbreaks caused by serotype A in eastern Asia since 1973. This incursion was followed by second introduction of FMDV in April, 2010, in this case serotype O (O/SEA/MYA/98). South Korea was declared free without vaccination by the OIE on 27^th^ September, 2010 after implementing a stamping out policy [79]. The third incursion, due to O/SEA/MYA/98, took place in November, 2010 and then spread throughout the country. Initially, a national stamping out policy was implemented for all animals on farms with FMDV infected animals. However, a nationwide vaccination-to-live policy was adopted later using emergency vaccination. Vaccination was effective in controlling the disease. A total of 3.48 million FMD susceptible animals were culled [80]. Similarly, Japan was hit by FMD ten years after the previous outbreak [81], FMDV type O, belonging to the MYA-98 lineage within the SEA topotype, was detected on 20^th^ April, 2010 at a beef feeding farm in southern Japan. The disease spread to the surrounding areas. Emergency vaccination was started on 22^nd^ May, 2010 within the infected zones. All the vaccinated animals were subsequently destroyed. During the three-month FMD epidemic, a total of 292 infected farms were detected and 290,000 animals were destroyed as a control measure [82]. The VP1 sequence data indicate that mainland Southeast Asia is the source of FMDV serotypes O and A in Eastern Asia [80,83].

In 2000, the O-PanAsia virus was detected in Uzbekistan, Mongolia, Armenia, Georgia and Russia and then in 2001 in Kyrgyzstan and during 2001–2003 in Tajikistan [84]. In 2000, the virus spread to KwaZulu-natal Province in South Africa. This was the first recorded outbreak in that country due to serotype O and the first since 1957 in this region of Southern Africa [85,86]. The outbreak was attributed to the feeding of swill to pigs from a ship which had originated from Asia. More recently, outbreaks due to SAT1 FMDV were reported in 2011 and further FMD outbreaks due to SAT2 have occurred in South Africa in 2012 while outbreaks due to SAT1 have been reported again in 2013 [87].

In 2001, the PanAsia virus was introduced into Europe where it caused disease outbreaks in the UK and was then spread to Ireland, France and the Netherlands. The outbreaks of FMD in United Kingdom in 2001 were the first since a single case in 1981 [88]. Before the 1981 case, the UK was hit by a major outbreak of FMD in 1967 and 442 000 animals were slaughtered to achieve eradication of the disease [89]. The 2001 outbreaks were controlled using a stamping out policy in which 6.5 million infected and in-contact animals were killed. The total economic losses due to the 2001 outbreak were estimated at between USD 12.3-13.8 billion [90]. Another outbreak hit the UK in 2007 and it was later identified that the disease was due to escape of virus from either the vaccine production facility or the Institute for Animal Health, both sited in Pirbright [8].

Currently, the O-PanAsia-II^ANT-10^ strain is responsible for extensive outbreaks in the whole Middle East and South Asian region. This strain was responsible for FMD in Pakistan in 2009, which spread westwards and caused disease in Turkey, Israel, Libya and Bulgaria [12,91]. Bulgaria experienced an outbreak of FMD at the end of 2010, the first case of the disease was detected in wild boar. FMDV serotype O virus strain O-PanAsia-II^ANT-10^ was found to be responsible for the outbreaks at 11 separate sites which occurred in two separate phases [12,92]. The country had remained free of FMD for 14 years since its previous outbreak in 1996. The disease also affected cattle, buffalo, sheep and goats. A stamping out policy was used and a total of 1372 animals were destroyed. Seropositive wildlife were detected near the outbreaks but the disease has not been maintained by them since no new cases of FMD were detected after April, 2011 and the country was declared free of FMD in July, 2011 [87].

## 7. FMD virus pools

**Figure 2:**
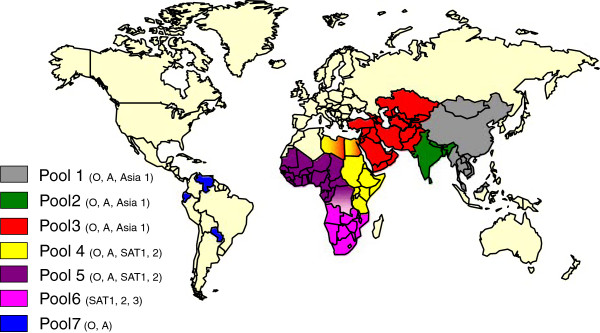
**Geographical distribution of seven pools of foot-and mouth disease viruses.** Serotype O FMDV is the most widely distributed serotype of the virus (in 6 of the 7 indicated virus pools) whereas, in contrast, SAT3 is only present in pool 6 (within southern Africa). The Asia-1, SAT1 and SAT2 serotypes also have quite limited geographical distribution. However, individual countries can have multiple serotypes in circulation at the same time and hence it is necessary to be able to determine which serotype is responsible for an outbreak if vaccination is to be used. Countries which are normally free of the disease (marked in yellow) can still suffer incursions of the virus which can have high economic costs.

Despite the propensity and opportunities for spread of FMDVs into new regions, comparison of the VP1 coding nucleotide sequences reveals a tendency for similar viruses to recur in the same geographical area. This tendency apparently reflects some degree of ecological isolation, likely reflecting patterns of animal movement and trade or specific wildlife reservoirs (e.g. African buffalo) within a region. Based on genetic and antigenic analyses, FMDVs throughout the world have been sub-divided into seven regional pools [93,94]. Certain countries share viruses belonging to two different pools, for example, Egypt and Libya (Figure 2). Virus circulation and evolution within these regional virus pools result in changing needs for appropriate vaccine selection.

## 8. Progressive Control Pathway for FMD

In response to the repeated epidemic events and requests for assistance by affected and at risk countries in West Eurasia, FAO/EuFMD convened a meeting of 14 directly affected countries in Shiraz, Iran in 2008 at which a long term regional approach for progressive control of FMD in the region was developed, known as the West Eurasia Regional Roadmap [95]. It was the first time that a Progressive Control Pathway for FMD (PCP-FMD) was established to determine national progress and to develop national and regional actions plans and support. Several FAO projects supported both national and regional PCP activities, such as improved FMD laboratory networking (WELNET) and epidemiological support. The activities implemented since the 2008 meeting have allowed the detection of three epidemics of regional (West Eurasian) significance in the past 4 years. These epidemics were caused by the serotype A-Iran-05^BAR-08^ sublineage in 2008 [9], the type O PanAsia-II^ANT-10^ strains in 2009–12 [12,91,92], and the serotype Asia-1 (Group-VII) viruses [56] of 2008–12, all of which involved east to west migration of the virus and to some extent involved Central Asian countries. The rapidity and frequency of incursions in the past 4 years presents a major problem for disease control, particularly when the strains involved are poorly matched to the routinely used vaccines [9,56].

**Figure 3:**
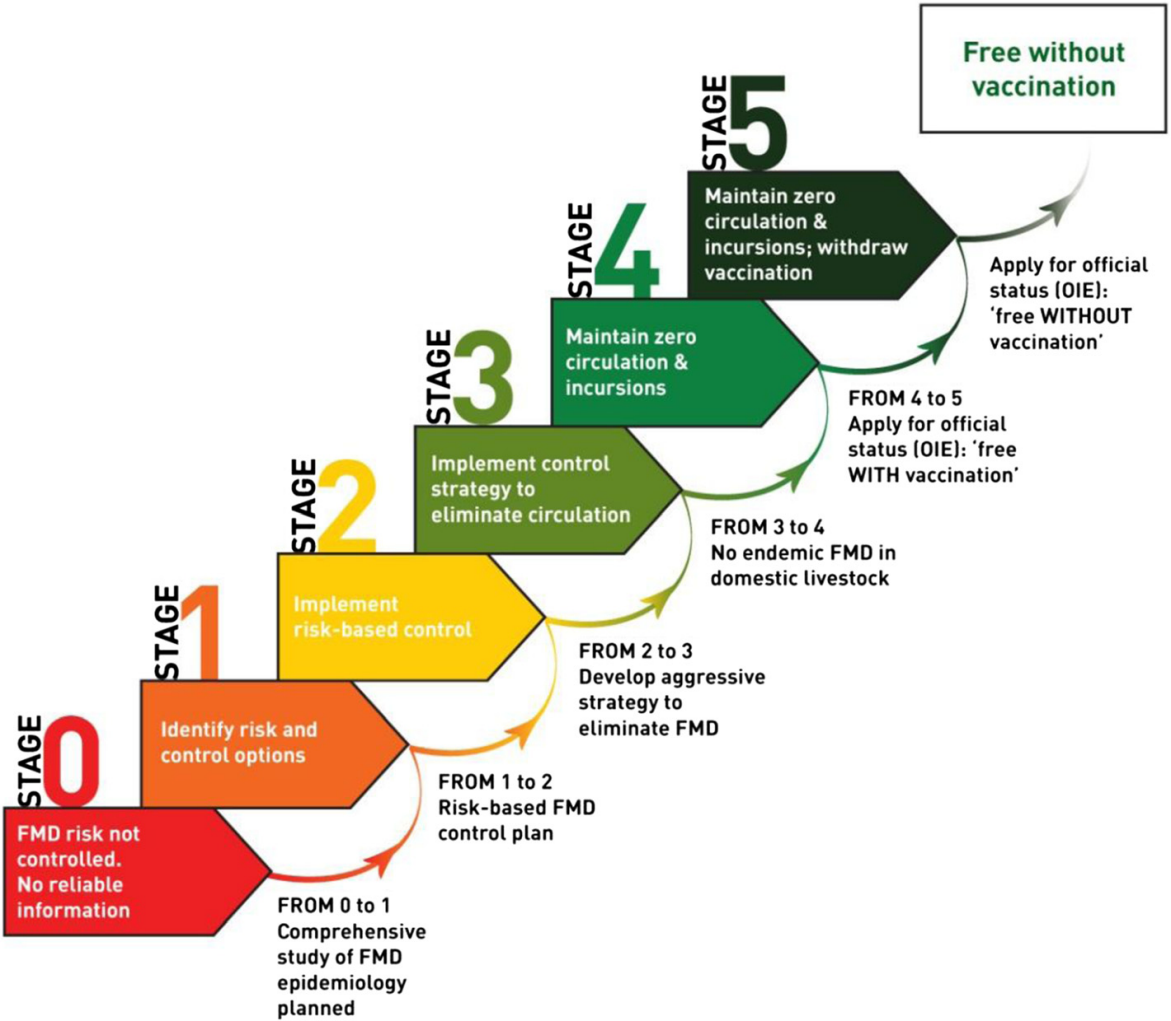
**The FAO/EuFMD/OIE Progressive Control Pathway for FMD.** The status of countries on the PCP-FMD is evaluated according to defined criteria. Countries with endemic disease are in stages 0 to 3 while countries with no endemic disease within livestock are at stage 4 or above. The image was kindly supplied by EuFMD.

The PCP-FMD has now been adopted as a joint tool between FAO/EuFMD/OIE [94]. This pathway includes a set of criteria to assist FMD endemic countries to progressively reduce the level of FMDV circulation and to mitigate the impact of FMD [96]. The PCP-FMD consists of a set of FMD control activity stages (Figure 3). Each stage has well-defined outcomes which can be achieved through a variety of activities. The specific activities required to achieve the outcomes have, however, not been prescribed. This non-prescriptive approach has the advantage that each country can adopt an approach according to its national/regional requirements and capabilities to achieve the outcomes. Understanding the “local” epidemiology of FMD and the active monitoring of the virus circulation are the foundations of the PCP-FMD and activities to meet these requirements are required in all stages [96]. Determination of the factors responsible for maintenance and spread of the disease and knowledge about circulating subtypes of FMDV are essential for effective control of the disease.

ELISAs for detection of antibodies to FMDV NSPs are generally used to monitor virus circulation as this test can discriminate between vaccinated and infected animals [44,45] if purified vaccines are used. The interpretation of NSP test results is, however, complicated in FMD endemic countries, where animals can be exposed to multiple serotypes and where non-purified vaccines are frequently used. Moreover, anti-NSP antibodies can persist for a long period [46] and do not necessarily indicate recent FMDV infection. Inadequate laboratory diagnostic capacity and its implications for the PCP-FMD in Eastern Africa have been described recently by Namatovu et al. [97]. In order to monitor FMDV circulation effectively, each participating country should, therefore, have sufficient FMD diagnostic and surveillance capacity. Progression along the PCP is based on an annual assessment of evidence-based activities related to FMD epidemiology and control measures undertaken by the country. The organizational infrastructure of each country is required to be progressively strengthened to ensure that the activities required to monitor and control FMD are implemented [98].

Countries where the disease is endemic with no reliable information on the disease status, are classified as in stage 0. In order to move from stage 0 to 1, a comprehensive study on the epidemiology of FMD is required to be planned [96]. No country in the West Eurasian region is now in stage 0 [94], the majority of the countries are presently in stage 1 and are expected to enter stage 2 by the end of 2013, whereas the Turkish Thrace region is currently in stage 4. Stage 1 assists in identifying appropriate control options. Countries in stage 1 are in the process of developing their control strategies in at least one animal production sector based on a comprehensive assessment of the epidemiology and control options. Progression from stage 1 to 2 requires a risk based FMD control plan. Thus stage 2 involves the implementation of the chosen policy. Countries in stage 2 have implemented a risk-based FMD control strategy that aims to reduce disease in at least one animal production sector. In order to move from stage 2 to 3, an aggressive strategy to eliminate FMD needs to be developed. Countries in stage 3 have adopted a control plan to progressively reduce/eliminate virus circulation in at least one region/production system [96]. This requires very significant national capacity and ongoing investment including the ability to ensure maintenance of sufficient herd immunity in critical populations to prevent FMD virus circulation.

Moving to stage 4 requires that FMD is controlled to an extent that it is not endemic in domestic livestock. If a country decides to continue along the FMD-PCP to stage 4 and beyond, it may ask the OIE for endorsement of its national FMD eradication program. Progression to stage 4 would thus indicate attaining officially recognized FMD free status with vaccination by the OIE for the whole or part of the country. Countries in stage 4 have maintained zero circulation with no incursions of FMD [96].

Vaccination plays a vital role in controlling FMD for countries in stages 2–4. Normally vaccine quality control is determined by the producer and vaccine batches are only released when they pass the quality control parameters. However, lack of maintenance of the cold chain before, during or after transport/importation may reduce the vaccine efficacy, even if the vaccine initially contained sufficient payload of serotype(s), matching with the circulating strain(s). Furthermore, as the dose–response relationship in FMD vaccination is influenced by the serotype and type of adjuvant present in the vaccine [99], both the antigen pay-load and the quality of the adjuvant in formulated vaccine need to be established. An independent quality control of the formulated vaccine is therefore necessary for effective control of the disease [100]. Vaccination alone may well not be able to contain the disease unless it is coupled with restrictions on animal movement. Control of animal movements is, however, complicated by many factors including social customs [52], religious festivals [17], trade of animals in live animal markets [10] and both formal and informal animal movement. Progression from Stage 4 to 5, and from Stage 5 to Pathway completion, would be through the existing official OIE recognition processes of freedom from FMD with or without vaccination, respectively.

In order to target resources available for surveillance and control of the disease, each stage of the PCP requires risk assessment and risk management activities. Socio-economic approaches including impact assessment, value chain analyses and cost-benefit analyses of intervention play an important role in animal disease control. However, integration of these approaches within animal disease control strategies is still in its infancy and remains a challenge.

The PCP-FMD approach has now been adopted for different regions including the West Eurasia roadmap 2020 (since 2008), the 2020 Roadmap for FMD Control in South-East Asia and China (SEACFMD), Southern Africa Roadmap (since 2011), Eastern Africa Roadmap (since 2012) and the Plan Hemisférico de Erradicación de la Fiebre Aftosa (PHEFA) for South America [94]. Thus the PCP is expected to form the backbone of the global FAO/OIE strategy for the control of FMD.

The West Eurasia roadmap 2020 projects that this region will reach stage 3 by 2020. The annual assessment of individual countries within West Eurasia, however, shows that the majority of countries did not reach the expected earlier stages at the projected times. It therefore seems unlikely that all the countries within West Eurasia will reach stage 3 by 2020.

## 9. Conclusions/recommendations

Despite considerable information being available about the virus, the disease and vaccines, FMD remains a major threat to the livestock industry world-wide. New sublineages of FMDV continue to evolve to produce novel strains which sometimes break through vaccine-induced immunity and can result in major epidemics. This warrants the need for continued surveillance, vaccine matching and vaccine quality control. Vaccination alone is unlikely to control the disease unless it is coupled with animal movement control. Animal identification systems and animal movement controls are therefore also needed to be in place for effective control of the disease.

The majority of countries in Asia and Africa, where the disease is endemic, are deficient in knowledge about the circulating sub-types of FMDV due to deficiencies in submitting outbreak samples to reference laboratories. The samples originating from these countries are often not collected in systematic way and are therefore not necessarily representative of the particular production systems/areas for which the PCP is intended. Each country must build capacity in diagnostics, epidemiology and economics in order to improve the information on the nature of FMDV circulation in that specific environment. Many of the countries participating in the PCP do not have the capacity to design and implement appropriate studies for determination of risk factors and for carrying out risk assessment and socio-economic studies. Furthermore, the non-descriptive approach in the PCP, in which the outcomes are defined but the means to achieve the outcomes are not specified, can itself be a hurdle in identification of risk factors for a particular environment/region. Therefore, these countries should be supported by helping them to design appropriate studies for determination of the relevant risk factors and for conducting risk assessment, risk management and socio-economic studies. The laboratory data should be combined with field epidemiological information for meaningful interpretation. Regional cooperation including timely information on the FMD outbreaks particularly in border areas, timely data/information sharing and simultaneous adoption of control measures are required for effective control of this transboundary disease. Early detection and early responses should be prepared in order to contain the disease effectively. Sufficient quality and quantity of assured FMD vaccines with matching serotypes needs to be made available for inducing sufficient herd immunity for further progression from stage 3 to stage 4 and preparations to achieve this can take some time.

## 10. Competing interests

The authors declare that they have no competing interests.

## 11. Authors’ contributions

SMJ conceived the idea, compiled the information and drafted the paper, GJB critically reviewed and revised the paper. Both the authors agreed to the final version of the manuscript.
